# Early cervical cancer screening: The influence of culture and religion

**DOI:** 10.4102/phcfm.v15i1.3776

**Published:** 2023-01-25

**Authors:** Fungai Gutusa, Lizeth Roets

**Affiliations:** 1 Department of Health Studies, College of Human Sciences, University of South Africa, Pretoria, South Africa

**Keywords:** Culture, stigma, religion, early screening, cervical cancer

## Abstract

**Background:**

Screening for cervical cancer at an early stage is essential for providing women with a better chance of receiving effective treatment for both precancers and cancer. Delaying screening until cancer has advanced can be detrimental, resulting in late presentation of cervical cancer and, as a result, cancer metastasis.

**Aim:**

The purpose of this study was to assess the extent to which culture and religion influence early cervical cancer screening in women.

**Setting:**

The research was conducted in one of the rural districts in Manicaland Province of Zimbabwe.

**Methods:**

A qualitative exploratory and contextual design was utilised, and data were gathered by means of semistructured interviews. At 17 semistructured interviews, data saturation was reached and further data collection terminated. Data were thematically analysed.

**Results:**

Five themes that described participants’ perceptions on culture and religion as barriers to early cervical cancer screening emerged from the data. These included a lack of knowledge, stigmatisation, cultural beliefs and values, religion and a lack of resources. These all negatively affected participants’ motivation to seek early screening services.

**Conclusion:**

According to the study findings, culture and religion constitute impediments to early cervical cancer screening for rural women. Interventions that encourage screening, such as targeted health education and health promotion materials, must consider cultural and religious views if behaviour change in diverse groups is to be accomplished.

**Contribution:**

The study has the potential to inform Zimbabwean health policy and contribute to prospective interventions or health education that encourage women to attend early cancer screening.

## Introduction

Cancer of the cervix accounts for over 22% of all cancers in sub-Saharan Africa,^1^ making it the fourth most common cancer among women worldwide, and is a major threat to public health.^2^ More than 60% of all cervical cancer cases are diagnosed late,^3^ contributing to women’s high rates of illness and death in resource-constrained settings.^4^ According to reports, the prevalence of cervical cancer in Zimbabwe is 35 per 100 000 women, whereas the global average is 15.^5^ Moreover, the country records almost 2270 new cases each year and approximately 1451 annual deaths.^5^ One of the main causes of cervical cancer is exposure to the sexually transmitted human papilloma virus (HPV), as untreated HPV can cause precancerous cells to develop into cervical cancer.^6^

Cervical cancer screening reduces a woman’s risk of developing the condition by approximately 80%.^4^ In 2011, Zimbabwe’s Visual Inspection with Acetic Acid and Cervicography (VIAC) programme for screening cancer of the cervix was implemented in over 105 healthcare facilities across the country.^7^ The screening programme has obstacles, including a lack of national procedures and regulations, as well as a lack of coordinating mechanisms.^8^ The healthcare professionals have been left with the onus to encourage women to be screened for cancer of the cervix because there is not an established government policy.^9^

Cervical cancer knowledge, level of education and certain cultural and religious beliefs have been found influencing women’s use of cancer screening programmes in other developing countries such as Kenya.^10^ Cultural and religious beliefs affect how people perceive health information and might function as a barrier between the healthcare professional and the patient;^11^ therefore, healthcare practitioners must be aware of the cultural and religious views of the communities in which they operate.^10^

According to studies completed in other African countries, namely Nigeria, Kenya and Botswana, women’s participation in cervical cancer screening programmes is low.^12^ The studies have also highlighted that there is a direct influence of culture on the provision of healthcare for chronic diseases.^12^ Culture influences a person’s knowledge and perception of cancer and cancer screening, contributing to low participation in cervical screening programmes.^13^

The purpose of this study was to describe the influence of culture and religion on a woman’s decision to utilise services for early cancer screening. Recommendations that may encourage women to access cervical screening opportunities were highlighted.

## Research methods and design

### Study design

A qualitative exploratory and contextual design was chosen for the study,^14^ as this was appropriate to capture how culture and religion can influence the decision to go for early cervical cancer screening.

### Setting

The study took place at a rural district hospital within Manicaland Province, one of the 105 health facilities in Zimbabwe that provide VIAC screening services.^15^ Zimbabwe is located in the southern part of Africa and is divided into 10 administrative provinces and 59 districts. The total population is approximately 14.9 million, and women represent 52% of the total population.^16^ The hospital was purposively selected, being a referral hospital for rural and peri-urban communities in the district and the only hospital in the district that offers cervical cancer screening daily.

### Study population and sampling strategy

The health centre where the study took place was randomly selected from the 46 health centres in the district. This study employed a convenience nonprobability method. Participants were chosen based on their proximity to the researcher and willingness to participate.^17^ Women between the ages of 18 and 45 (the reproductive age group), women who went to the selected rural district hospital for reproductive health services and women who lived in the health centre’s catchment area and who volunteered to participate in the study were included. Data were gathered until data saturation was reached, that is, until no new data or themes emerged.^18^

The gatekeeper (district medical officer) provided the contact information of eligible women attending reproductive health services (potential participants) to the researchers. Participants were provided with an information letter inviting them to volunteer to take part in the study. The researcher explained the study’s purpose to the participants in a clear and concise manner. Before the in-depth interviews commenced, participants were required to provide voluntary informed consent in writing. Seventeen women, ranging from 18 to 45 years, participated. The mean age was 29 years.

### Data collection

Data were collected using in-depth semistructured interviews, and interviews took place in September 2018. The data collection and concurrent analysis continued until 17 interviews were completed and viable topics were exhausted. Field notes were taken and the interviews audio-recorded. Each interview lasted between 45 min and 60 min and was conducted in Shona, Ndau and/or English, depending on which language the participant understood and was comfortable conversing in.

The interviews took place in quiet consulting rooms labelled ‘do not disturb’, away from patients’ waiting areas. The consulting rooms meant that there was no interruption and that the participants had privacy. This also allowed the researcher and participants to engage more in the interview process. Data were collected until the interviews failed to provide any new information, thus until saturation.^19^

### Data analysis

Thematic analysis was carried out, a dynamic method that allowed flexibility to find, analyse and interpret the large data sets and sort them into broad themes.^20^ Emerging ideas were scrawled in the margins of transcriptions, and similar thoughts were categorised. The authors applied all six of the steps suggested for a thematic analysis, namely familiarisation, coding, generating themes, reviewing themes, defining and naming themes and writing up.^17^

The authors began by transcribing the audio-recorded interviews, taking preliminary notes and going over the data several times to familiarise themselves with it. This was followed by the creation of initial codes from the data, where all data aspects were coded in a systematic manner. Themes and patterns were identified among the codes after they were categorised, and those that shared a similar meaning were grouped.^14^ To ensure that the themes were useful and depict an accurate representation of the data, the themes were compared with the original data set. The themes and categories were defined and named, and the analysis ended with the write-up and description of the research findings according to the identified themes.

### Ethical considerations

The Health Studies Research Ethics Committee at the Department of Health Studies of a large university in South Africa (reference number HSHDC/655/2017), the Medical Research Council of Zimbabwe (reference number MRCZ/B/1319) and the partnering hospital provided ethical clearance, while the district medical officer and the district nursing officer provided gatekeeper approval. The interview guide did not contain the real names of the participants to ensure that the interview data could not be linked to any individual; thus, confidentiality was maintained throughout the data collection process. Before data collection commenced, all respondents signed informed consent letters.

## Results

The participants’ ages ranged from 20 to 45, with most women (*f* = 29%) between 31 and 35. Six women were older than 35. Early screening for cervical cancer should be routine care for people between the ages of 35 and 44, because this is the age group at risk for cervical cancer.^21^

It is known that a woman’s marital status, spousal support, trust issues between spouses as well as stigmatisation by spouses affect their decision to go for cervical cancer screening.^21,22^ In this study, 10 participants were married (*f* = 58.8%), 3 were single (*f* = 16.4%), 1 (*f* = 5.9%) was divorced and 3 (*f* = 16.4%) were widowed.^23^

As illustrated in Table 1, 5 broad themes and 12 categories were derived during the analysis. These themes were: (1) a lack of knowledge, (2) stigmatisation, (3) cultural norms and beliefs, (4) religion and (5) a lack of resources.^23^

**TABLE 1:** Themes and categories.

Themes	Categories	Subcategories: Direct quotes
Lack of knowledge	Symptoms	‘You cannot just find yourself at the hospital whilst you are not sick. As long as one is not feeling well there is no need to go to the clinic.’ ‘If someone is not feeling well and they suspect they have cervical cancer, then they should be screened but if they are fine there will be no need.’ ‘I am not sure if it is really important to get screened, but I think as long as one is having vaginal bleeding and foul-smelling vaginal discharges, they should be screened.’ ‘I will not go to the clinic when I am not sick, it is a waste of time. It’s only those who are sick who needs a nurse or a doctor.’
	Incurable disease	‘I don’t see any reason of going at the clinic because I was told that Cancer is a killer disease that cannot be cured.’ ‘What is the use of getting to know that I have a disease when the nurses cannot cure the cancer disease?’ ‘We grew up being told that cervical cancer cannot be treated and people with cancer eventually die from the disease.’
	Side effects	‘My friend told me that they insert a metal object in the cervix which they scrap with an object during screening.’ ‘I fear contacting other diseases such as HIV and AIDS as I have heard that most doctors that have been treating patience end up catching the disease.’
Stigmatisation	Attitudes	‘Cervical cancer is disease which many people including me do not discuss about with friends and neighbours because people may gossip and laugh thereafter.’ ‘Every woman should get screened in time to ensure that they get treated early if any signs of cancer is detected.’ ‘I am not ready now; I have other important things that I need to be attending to.’ ‘People in this community believe that if a person is said to have such a disease is a curse because bad luck falls upon them due to doing some bad things such as sinning.’
Cultural beliefs and values	Trust and embarrassment	‘The only problem I got was when I was told to remove my clothes, but later I realized they were fellow women. I looked at them, I became strong and removed the clothes.’ ‘Cervical cancer is considered to be a sexually transmitted disease and having such a disease will lead to loss of trust for the wife by her husband who may think that the wife was sleeping around with other men.’
	Traditional medicine	‘My grandmother when she got cancer visited an herbalist who resides in our village.’ ‘I don’t see any reason for screening at a health centre because cancer is only treated by herbs and not the modern medicine.’
	Husbands’ involvement	‘My husband decides whether I should go for screening or not.’ ‘If I want to go to the clinic, I seek permission from my husband and if he doesn’t want me to go for cancer screening, I have to be obedient.’ ‘Men are not comfortable with their women being checked their private parts.’ ‘Our culture does not allow women to go for medical examination alone without any male members of the family, this means that I can go to the clinic for screening only when my husband is around and willing to accompany me.’ ‘Our men do not want anyone who goes to check especially the private parts of the body, they will become suspicious if you start talking of private parts examination and this can destabilize a marriage if you do without their permission.’ ‘My husband may divorce me if he knows that I have cervical cancer.’ ‘This disease is associated with cheating in marriages, therefore getting screened will cause male partners to marry another healthier woman.’
Religion	Spiritual leaders	‘Some spiritual leaders opt for prayers and in case a person is infected they will be prayed for, and they get healed.’ ‘My pastor always gives me holy water every when I go to church, and I am sure that will help me not to suffer from cervical cancer.’ ‘There is discussion held mostly in Pentecostal churches where people interact and teach about the importance of cervical cancer screening.’ ‘There is nothing as such hidden from our church as everything is revealed to them through the prophets. The prophets will pray for us so that we do not contract diseases including cancer.’
	Religious beliefs	‘I believed cervical cancer is a disease that is unavoidable, and it is in only in God’s nature in control.’ ‘People in this community believe it’s a curse to have such a disease as cancer and therefore cleansing by holy water and prayers is needed.’
Lack of resources	Policies	‘There is lack of cervical cancer testing services that motivate us in the rural areas to seek cervical cancer screening.’ ‘Cervical cancer screening should be done for free in all clinics whether government of private.’ ‘We need this educational forum so that we can understand. It is the same as when HIV started, there was little information given, but when they started laying it out then people started to understand.’
	Lack of equipment	‘Sometimes when you go to the clinic they say some things are not there (speculums) so we have to go and check with bigger hospital.’ ‘The clinic is far, and also long queues and also sometimes they say they don’t have tools.’
	Lack of human resources	‘Nurses are few compared to the patients in the rural areas, they fail to meet the demand.’ ‘I once went to the clinic and I came back before screening after waiting the whole day without being attended to.’

HIV, human immunodeficiency virus; AIDS, acquired immunodeficiency syndrome.

## Discussion

Culture and religion were found to be obstacles to early cervical cancer screening. The findings also demonstrated that other variables influence women’s decision to undergo early screening for cervical cancer.

### Lack of knowledge

Screening for cervical cancer is a topic about which most women have little knowledge, and they often present for treatment following the onset of cervical cancer symptoms,^24,25^ which is thus too late. The research findings revealed that women delay screening for cervical cancer because they are unaware of the value of early detection. Similar to the present study’s findings, Tapera et al.^15^ reported that poor screening behaviour was a result of a lack of awareness about the risks and causes of cervical cancer.

Delaying screening until cancer has progressed to an advanced stage can have catastrophic effects, as it can lead to cancer metastasis.^26^ Linked to the lack of knowledge is the absence of symptoms in the early stages of the disease, which may be detrimental when deciding to utilise early cervical cancer screening services.^27^ One of the reasons, according to the study participants, for why women were reluctant to seek cervical cancer screening services, particularly in its early stages, was the absence of symptoms. A participant confirmed that women do not undergo cervical cancer screening if they do not have any symptoms:

‘You cannot just find yourself at the hospital whilst you are not sick. If one is not feeling unwell, there is no need to go to the clinic.’ (Particpant 5)

Because of a lack of knowledge about the disease, participants reported not knowing that cervical cancer is a preventable condition. Because of this lack of awareness, women were less likely to seek early cancer screening. A participant explained:

‘I do not see any reason of going to the clinic because I was told that cervical cancer is a killer disease that cannot be cured.’ (Participant 7)

Participants allegedly believed that the cervical cancer screening method was painful, and their fear of discomfort stopped them from getting screened early. A participant claimed:

‘My friend told me that they insert a metal object in the cervix which they scrap during screening, and she said the object is very painful.’ (Participant 9)

The absence of pain or symptoms does not always mean there is no disease; cancer of the cervix progresses slowly, and the symptoms only occur after the cancer has progressed to a level where treatment is difficult.^28^ Several of the symptoms of cervical cancer, including vaginal discharge and bleeding, were unknown to the participants, possibly resulting in poor patient outcomes.

### Stigmatisation

Cervical cancer is associated with HPV, a sexually transmitted illness, and people equate HPV and cancer with sexual promiscuity.^9^ Participants noted that stigma associated with perceived incurable conditions, such as cervical cancer and HIV, was a barrier to early screening.^29^ Participants reported that women were afraid of stigmatisation. This finding was supported by Nyblade et al.,^30^ who mentioned that women’s concerns about the reactions of their friends, family and neighbours discouraged them from seeking early screening. The participants’ narratives, as recounted below, emphasised their fear of stigma:

‘Cervical cancer is disease which many people including me do not discuss about with friends and neighbours because people may gossip and laugh thereafter.’ (Participant 15)

Participants with some awareness of cervical cancer said they were not embarrassed to talk about cervical cancer screening with their acquaintances. However, only three of the participants said they would go for cancer screening.

### Cultural beliefs and values

Culture has been found to be a hitch to early screening for cancer of the cervix, as studies revealed.^20^ Feeling embarrassed to undress in front of health workers and over-reliance on traditional medicine are other common factors preventing women from screening for early signs of the disease.

Because of a lack of awareness regarding the benefits of early screening among men, they often postpone or refuse to allow their wives to receive the service. Women therefore delay going for screening because they need approval from their husbands:

‘If I want to go to the clinic, I seek permission from my husband, and if he doesn’t want me to go for cancer screening, I have to be obedient.’ (Participant 12)

Participants expressed embarrassment at having to undress in front of nurses, particularly when male nurses conducted cervical cancer screening. They expressed that undressing in front of strangers is an invasion of their privacy and indicated it was against their culture as was emphasised by participants:

‘The only problem I got was when I was told to remove my clothes, but later I realised they were fellow women. I looked at them, I became strong and removed the clothes.’ (Participant 6)

Modern science has proven that early screening for possible cervical cancer is crucial for the disease’s early detection, even before any symptoms appear. However, women often regard cervical cancer as an ailment that can be treated only with traditional medicine and herbs.^31^ Traditional medicine has long been utilised in various parts of Africa, such as Zimbabwe and Ethiopia, as complementary medicine for cancer treatment.^32^ Participants said they turn to traditional medicine when faced with cancer, but relying too heavily on it can result in women overlooking cervical cancer screening opportunities. As one participant stated:

‘My grandmother, when she got cancer visited an herbalist who resides in our village.’ (Participant 1)

Although it is documented that traditional medicine can be effective in the treatment of common ailments and cancer in general, an excessive reliance on it may delay cervical cancer screening, ultimately affecting patient outcome.^31^

### Religion

Cervical cancer screening can be hindered by religion, although some church leaders use their religious platforms to promote awareness and encourage their congregants to seek preventive services in general, which might be used to raise awareness about cervical cancer screening as well.^19^ Christian believers of the apostolic faith typically use holy water for disease prevention and as complementary medicine for treatment of cancer.^30^ Participants identified prophets and church leaders who protect them with prayers, believing that their faith will keep them from diseases such as cancer of the cervix:

‘My pastor always gives me holy water every when I go to church, and I am sure that will help me not to suffer from cervical cancer.’ (Participant 4)

The belief that diseases like cervical cancer are retribution for previous wrongdoings was mentioned as influencing women’s cervical cancer screening decisions. As one individual put it:

‘People in this community believe it’s a curse to have such a disease as cancer and therefore cleansing by holy water and prayers is needed.’ (Participant 7)

Onyenwenyi and Mchunu agree that religious doctrines can prevent members from accessing medical care such as cervical cancer screening at clinics and hospitals,^24^ ultimately affecting patient outcomes.

### Lack of resources

Governmental institutions such as public clinics and hospitals face equipment shortages, such as gloves, fluorescent lights, acetic acid and vaginal speculums, resulting in uneven service delivery.^33^ In this study, participants mentioned long distances to the nearest screening service facilities, inadequate number of nurses at clinics, as well as the unavailability of, among others, speculums used for vaginal inspection as some of the barriers preventing them from being screened for cervical cancer. Participants mentioned:

‘Sometimes when you go to the clinic, they say some things are not there [*speculums*], so we have to go and check with bigger hospital.’ (Participant 2)

Women expressed dissatisfaction when they went to clinics or hospitals for screening to find that there was no equipment available or that they had to wait in long queues because of the few nurses available to attend to them. A participant indicated that:

‘I went to the clinic, and I came back before screening after waiting the whole day in a long queue without being attended to.’ (Participant 10)

The strength of this research is the use of qualitative, in-depth, face-to-face interviews for data collection that allowed the researchers to obtain a wealth of information on why women delay seeking early cervical cancer screening, and this came from the perspective of the individuals. To answer the research objective, it was necessary to employ a qualitative research design. The limitation of this study is the inclusion of only one rural health centre in Mutasa District in Manicaland Province, which may not be a representation of all health centres in Manicaland Province.

### Recommendations

Based on the study findings, it will be advantageous to upscale preventive interventions to include expanding knowledge about cervical cancer and addressing attitudes towards screening, taking into account the diversity in beliefs and cultures of all, to motivate women to undergo early cervical cancer screening. This will facilitate improved access to cervical cancer screening services by women. A cross-sectional study to determine the accessibility of cancer screening services at all levels of healthcare must be conducted.

Research that focuses on development and management of cervical cancer using conventional medicine may be able to offer interventions and possible solutions for cancer prevention and treatment. Religious leaders’ influence on women’s decisions to undergo cervical cancer screening at an early stage should be explored.

## Conclusion

Women’s decisions to go for early cervical cancer screening are affected by a lack of information, a lack of adequate resources, as well as cultural and religious beliefs. Healthcare providers can facilitate the provision of health education materials concentrating on women’s health, including early cancer screening. Given the cultural and religious diversity of the population, those responsible for health promotion material must consider the communities’ cultural and religious perspectives.

To address women’s perceptions of cervical cancer screening, the Ministry of Health and Child Care (MOHCC) ought to provide vernacular and context-specific health education materials in the form of pamphlets and posters. Images and graphs containing information regarding the benefits of having early screening for cervical cancer should be utilised at all healthcare facilities, stores and other public areas, and they should be shared on social media.
